# Survival and clinicopathological significance of B7-H3 in bladder cancer: a systematic review and meta-analysis

**DOI:** 10.1186/s12894-024-01446-3

**Published:** 2024-03-11

**Authors:** Haohao sun, Fei Gao, Yuan Liu, Jianfeng Shao

**Affiliations:** 1 Department of Urology, Wuxi No.2 People’s Hospital (Jiangnan University Medical Center), Wuxi, 214002 China; 2 Department of Urology, Affiliated Wuxi People’s Hospital of Nanjing Medical University, Wuxi, 214023 China; 3 Department of General Surgery, Affiliated Wuxi People’s Hospital of Nanjing Medical University, Wuxi, 214023 China

**Keywords:** B7-H3, Biomarker, Bladder cancer, Clinicopathological characteristics, meta-analysis

## Abstract

**Background:**

B7-H3 has been implicated in clinical pathological features and prognosis across various cancer types, suggesting its potential as a cancer biomarker. Nevertheless, consensus remains elusive regarding its clinical-pathological and prognostic significance in bladder cancer. To address this gap, we conducted a systematic review and meta-analysis.

**Methods:**

We systematically searched PubMed, Embase, Web of Science, Cochrane, and CNKI databases from their inception up to October 6, 2022. We evaluated the literature’s quality using the Newcastle-Ottawa Scale. We performed meta-analysis using Review Manager 5.3 and STATA 12.0, synthesizing data and calculating odds ratios (ORs) or hazard ratios (HRs) with corresponding 95% confidence intervals (CIs).

**Results:**

After applying eligibility criteria and conducting assessments, we included data from 8 studies, encompassing 1622 bladder cancer patients. Bladder tumor tissues exhibited significantly elevated B7-H3 protein expression compared to normal bladder tissues. Elevated B7-H3 expression was notably associated with patient age, tumor infiltration, and recurrence in bladder cancer. However, no significant correlations were observed with other clinical characteristics. Our pooled HR analysis indicated no significant association between B7-H3 expression and overall survival in bladder cancer patients.

**Conclusion:**

Our meta-analysis unveils the complex role of B7-H3 in bladder cancer progression. It appears to be directly involved in tumor infiltration and recurrence but cannot definitively serve as a prognostic biomarker for bladder cancer. To validate these findings, further well-designed studies, encompassing larger sample sizes and diverse racial backgrounds, are warranted.

**PROSPERO registration:**

No. CRD42022364688.

## Introduction

Bladder cancer (BC) is a common cancer of the urinary tract, with an estimated 573,278 new cases and 212,536 deaths annually worldwide [1]. The disease is clinically classified into nonmuscle invasive bladder cancer (NMIBC)(Ta/T1) and muscle-invasive bladder cancer (MIBC)(T2-T4), based on different clinical progressions and prognoses. Despite advancements in current therapeutic methods, including surgery, radiation therapy, and chemotherapy, the 5-year overall survival (OS) rate remains unsatisfactory, particularly for advanced bladder cancer [2–4]. Therefore, finding a novel biomarker as an effective therapeutic target to improve the prognosis of bladder cancer patients is crucial.

Bladder cancer (BC) has been extensively studied for prognostic markers [5–9]. CTCs have emerged as potential prognostic markers in various cancers [10–12], including BC [13], but their detection and enrichment present challenges due to their scarcity and molecular heterogeneity [9]. FGFR3 mutation status is also linked to clinical prognosis, with better survival rates observed in patients with the FGFR3 mutation [14, 15]. However, agents targeting FGFR3 mutation are still in early clinical experiments, and their efficacy and safety remain uncertain [16–18]. Additionally, HER2 status has been associated with higher stage and grade and poor disease-specific survival, particularly in muscle-invasive and metastatic BC [19, 20]. However, HER2’s prognostic role in non-muscle-invasive BC is more debatable [8, 21]. Given these limitations and uncertainties, there is a need for improved prognostic biomarkers in BC to enhance clinical management and treatment decisions.

B7-H3 (CD276) belongs to the B7 superfamily of molecules and shows potential as a promising target for cancer treatment. The expression of the B7-H3 protein has been observed in various tumor tissues, including non-small cell lung cancer (NSCLC) and prostate cancer, and it is closely associated with tumor progression, metastasis, recurrence, and other adverse clinical features [22–27]. Its prognostic impact has been established in certain solid tumors, including clear cell renal cell carcinoma, breast cancer, and gastric cancer [28–30]. While several studies have explored the connection between bladder cancer and B7-H3 expression, the clinical-pathological and prognostic significance of B7-H3 in bladder cancer remains uncertain.

## Materials and methods

This meta-analysis adhered to the preferred reporting items for systematic reviews and meta-analysis (PRISMA) guidelines. The protocol for the overarching project has been published and registered with PROSPERO (registration No. CRD42022364688).

### Study search strategy

Two independent researchers conducted a search for published studies on B7-H3 expression and bladder cancer in multiple databases, including PubMed, Embase, Web of Science, Cochrane, and CNKI, up until 6 October, 2022. The search strategy employed the following keywords: “B7-H3” or “B7H3” or “CD276” and “bladder cancer*” or “bladder tumor*” or “bladder neoplasm*” or “bladder carcinoma*” or “BC” or “Urinary Bladder Neoplasms [MeSH].” Additionally, the reference lists of the retrieved studies were manually reviewed to identify any potentially relevant articles. There were no limitations regarding country, race, or language when conducting the study search.

### Inclusion and exclusion criteria

All included studies in this meta-analysis fulfilled the following inclusion criteria: (1) The study patients were diagnosed with bladder cancer by histopathology, and B7-H3 expression was precisely detected. (2) The study investigated the association between B7-H3 expression and clinicopathological features. (3) The expression level of B7-H3 was categorized into two levels: high or low. (4) Hazard ratios (HRs) for overall survival (OS) or disease-free survival (DFS) could be calculated from the survival curves. (5) If there were repeated studies, the most recent report was included in the meta-analysis.

The exclusion criteria were as follows: (1) Secondary research, such as reviews, meta-analyses, case reports, and conference papers. (2) Animal research studies. (3) Studies with insufficient information or conflicting data. (4) Duplicated studies.

### Data extraction and quality assessment

For each study, the following information was recorded: the first author’s name, publication year, sample resources, number of cases, age, gender, depth of invasion, histological grade, lymph node metastasis, and follow-up duration. In cases where specific data were not available, the hazard ratio (HR) of overall survival time was either collected or inferred from the Kaplan‒Meier curve. The required data were directly extracted or obtained from the survival curve using Engauge Digitizer 4.1 software to calculate the HR and 95% CI. Two independent observers (SHH and GF) independently performed the data extraction. The quality of the selected articles was assessed using the Newcastle-Ottawa Scale (NOS) criteria [31]. In instances where data could not be obtained from the literature, we considered the related data as missing.

### Statistical analysis

Statistical analysis was conducted using Review Manager 5.3 and STATA12.0 software. The relationship between B7-H3 expression and clinicopathological features or overall survival time of bladder cancer was assessed using pooled odds ratio (OR) or hazard ratio (HR) with 95% confidence interval (CI). The statistical significance of the OR and HR was determined by the Z-test and the corresponding P-value. Heterogeneity among the included studies was assessed using the I^2^ test with the corresponding P-value; if I^2^ ≥ 50% and *P* ≤ 0.05, significant heterogeneity was present, and the random effects model was used. Conversely, if I^2^ < 50% and *P* > 0.05, no significant heterogeneity was observed, and the fixed effects model was applied.

Publication bias was evaluated through Egger’s test and Begg’s funnel. Sensitivity analysis was employed to assess the reliability and stability of the meta-analysis results. A P-value ≤ 0.05 was considered statistically significant.

## Results

### Study selection

**Fig. 1:**
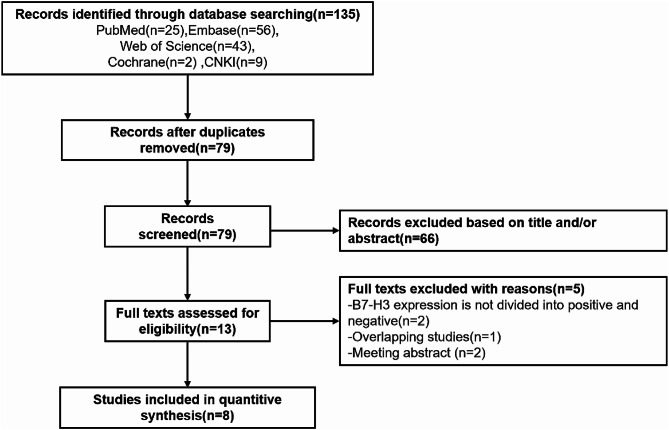
Flow diagram of study selection

Figure 1 illustrates the flow chart describing the study selection process. Initially, the search strategy identified a total of 135 studies. After removing duplicates, 79 records underwent screening based on title and abstract. Among them, 13 articles remained for full-text evaluation. Subsequently, 5 out of the 13 articles were excluded for the following reasons: two was a meeting abstract, one was a duplicate study, and two did not provide B7-H3 expression division. Ultimately, eight studies were included in this meta-analysis [32–39].

### Characteristics of the included studies

Table 1 presents the main characteristics of the eight eligible studies. The publication years ranged from 2014 to 2022, with seven studies published in English [33–39] and one in Chinese [32]. Four studies were conducted in China [32, 34, 35, 37], three in America [33, 36, 38], and one in Japan [39]. The sample sizes varied from 45 to 555, totaling 1622 participants. Among them, five studies [32–35] reported the correlation between B7-H3 expression and overall survival (OS), and all eight studies demonstrated an association between B7-H3 and clinicopathological features. Immunohistochemistry (IHC) was used in seven studies [32–38] to detect B7-H3 expression, while one study [39] used enzyme-linked immunosorbent assay (ELISA). All the eligible entries scored higher than six on the Newcastle-Ottawa Scale (NOS), indicating high methodological quality across all studies.

**Table 1:** Characteristics of included studies

Study	Year	Country	Study period	Detection method	case	control	language	NOS
Azuma	2020	Japan	2008–2013	ELISA	555	103	English	6
Boorjian	2008	America	1990–1994	IHC	314	146	English	6
Li	2017	China	-	IHC	45	45	English	8
Mahmoud	2022	America	1985–2019	IHC	81	-	English	8
Xu	2016	China	2008–2010	IHC	76	76	Chinese	7
Xu	2018	China	2005–2006	IHC	115	-	English	7
Xu’	2018	China	2004–2005	IHC	134	-	English	7
Xylinas	2014	America	1988–2003	IHC	302	50	English	8

IHC = immunohistochemistry; ELISA = enzyme linked immunosorbent assay

### The expression of B7-H3 in BC

**Fig. 2:**
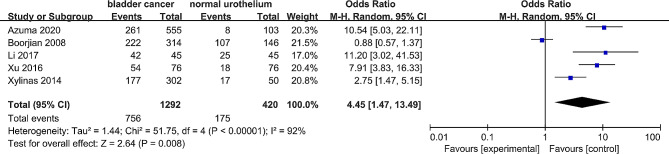
Forest plot of the association of B7-H3 expression between bladder cancer and non-tumor tissue samples

Data from five studies [32, 33, 37–39], including 1292 patients with bladder cancer and 420 nontumor tissue samples, demonstrated that B7-H3 expression was significantly higher in bladder cancer compared to nontumor tissue samples (OR = 4.45, 95% CI = 1.47–13.49, *P* = 0.008) (Fig. 2).

### Relationship between B7-H3 expression and clinicopathological features of BC

**Fig. 3:**
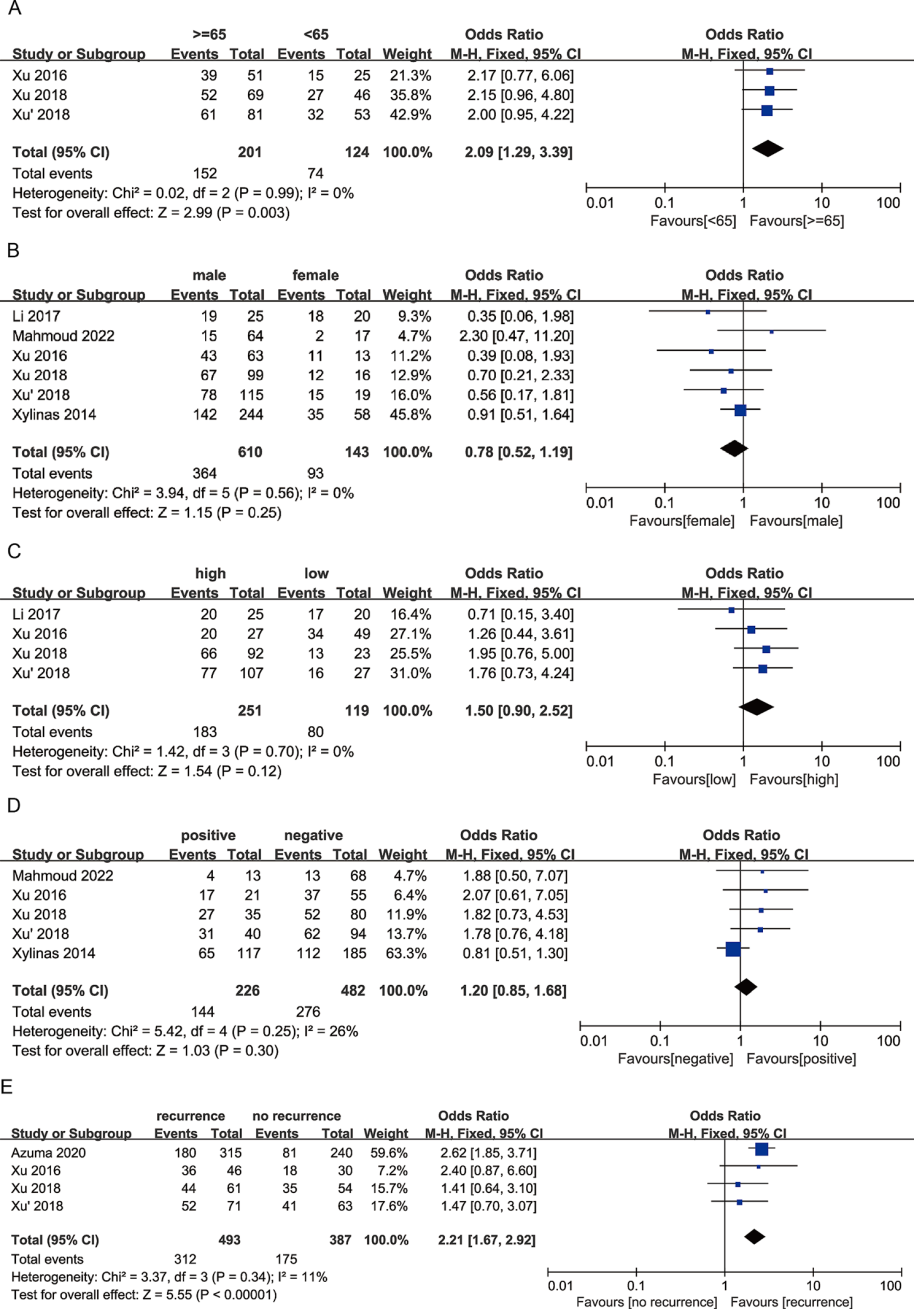
Forest plot of the association between B7-H3 expression and **A** age, **B** gender, **C** tumor grade, **D** lymph node metastasis, **E** recurrence

**Fig. 4:**
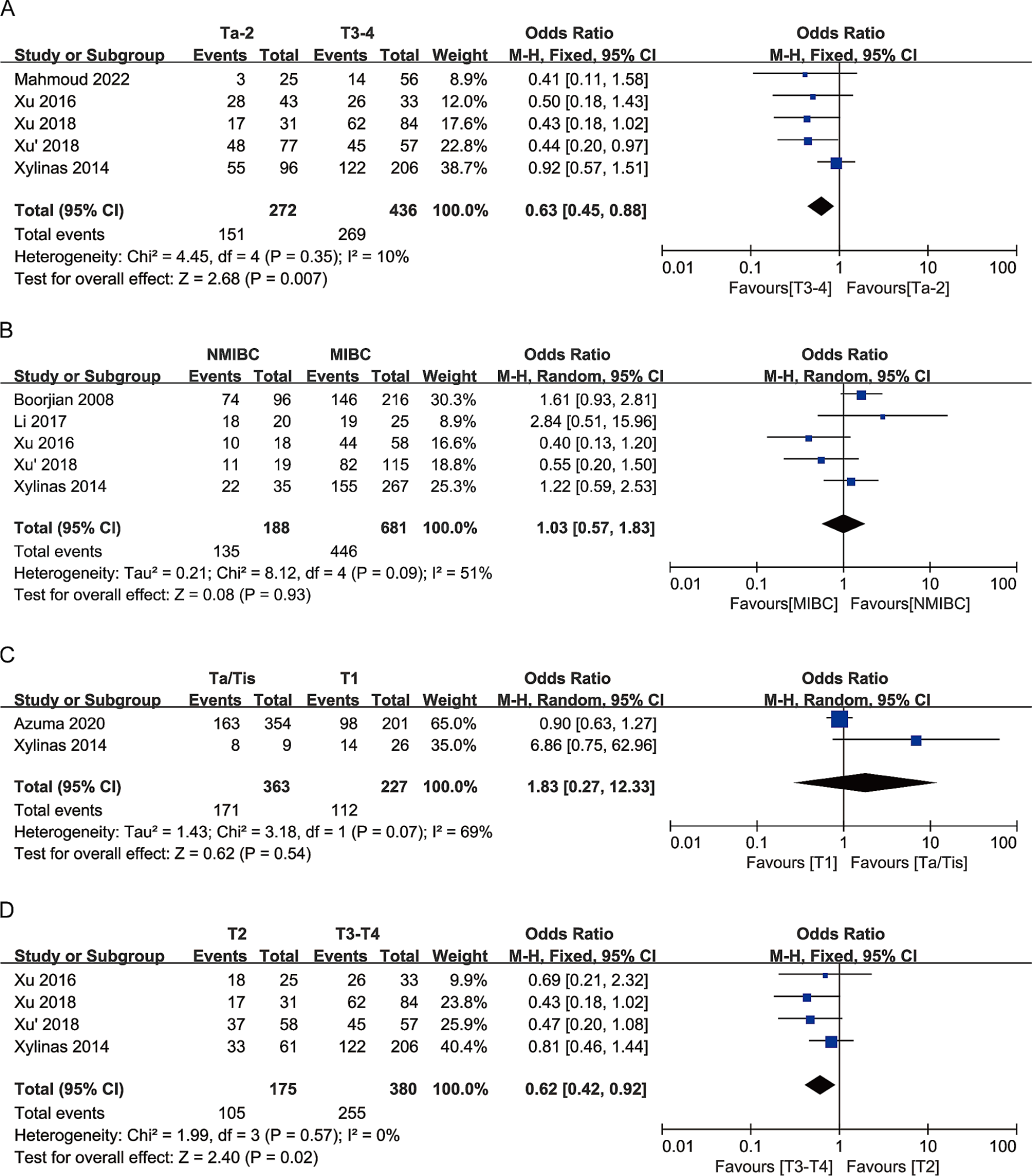
Forest plot of the association between B7-H3 expression and **A** tumor stage(Ta-2 vs. T3-4), B tumor stage(NMIBC vs. MIBC), **C** tumor stage in NMIBC (Ta/Tis vs. T1), **D** tumor stage in MIBC (T2 vs. T3-4)

In this meta-analysis, we compared the relationships between B7-H3 expression and clinicopathological characteristics, such as age, gender, T stage, tumor grade, recurrence, and lymph node metastasis, based on 8 studies (Table 2). The results of the meta-analysis indicated significant associations between high B7-H3 expression and age (Fig. 3A), higher tumor stage (Ta-2 vs. T3-4) (Fig. 4A), advanced tumor stage of muscle-invasive bladder cancer (MIBC) (T2 vs. T3-4) (Fig. 4D), and recurrence (Fig. 3E). The combined odds ratios (ORs) and 95% confidence intervals (CIs) were as follows: OR 2.09, 95% CI 1.29–3.39, *P* = 0.003; OR 0.63, 95% CI 0.45–0.88, *P* = 0.007; OR 0.62, 95% CI 0.42–0.92, *P* = 0.02; and OR 2.21, 95% CI 1.67–2.92, *P* < 0.00001, respectively.

However, we did not observe significant associations between B7-H3 and gender (Fig. 3B), higher tumor stage (NMIBC vs. MIBC) (Fig. 4B), tumor stage in non-muscle invasive bladder cancer (NMIBC) (Ta/Tis vs. T1) (Fig. 4C), tumor grade (Fig. 3C), and lymph node metastasis (Fig. 3D) in bladder cancer patients. The combined ORs and 95% CIs were as follows: OR 0.78, 95% CI 0.52–1.19, *P* = 0.25; OR 1.03, 95% CI 0.57–1.83, *P* = 0.93; OR 1.83, 95% CI 0.27–12.33, *P* = 0.54; OR 1.50, 95% CI 0.90–2.52, *P* = 0.12; and OR 1.20, 95% CI 0.85–1.68, *P* = 0.30, respectively .

**Table 2:** Results of subgroup analysis of the association between B7-H3 expression and clinicopathological parameters

Outcome of interest	Studies(n)	Patients	OR (95%CI)	P value	Model	Heterogeneity	
						I2(%)	P value
Age (≥ 65 vs. <65 years)	3	325	2.09(1.29,3.39)	0.003	Fixed	0	0.99
Gender (Male vs. Female)	6	753	0.78(0.52,1.19)	0.25	Fixed	0	0.56
Tumor grade (low vs. high)	4	370	1.50(0.90,2.52)	0.12	Fixed	0	0.7
LN metastasis (Positive vs. negative)	5	708	1.20(0.85,1.68)	0.3	Fixed	26	0.25
Depth of infiltration							
Ta-2 vs. T3-4	5	708	0.63(0.45,0.88)	0.007	Fixed	10	0.35
NMIBC vs. MIBC	5	869	1.03(0.57,1.83)	0.93	Random	51	0.09
NMIBC (Ta/Tis vs. T1)	2	590	1.83(0.27,12.33)	0.54	Random	69	0.07
MIBC (T2 vs. T3-4)	4	555	0.62(0.42,0.92)	0.02	Fixed	0	0.57
Recurrence vs. No recurrence	4	880	2.21(1.67,2.92)	< 0.00001	Fixed	11	0.34

LN: Lymph node

### The prognostic value of B7-H3 expression in patients with bladder cancer

**Fig. 5:**
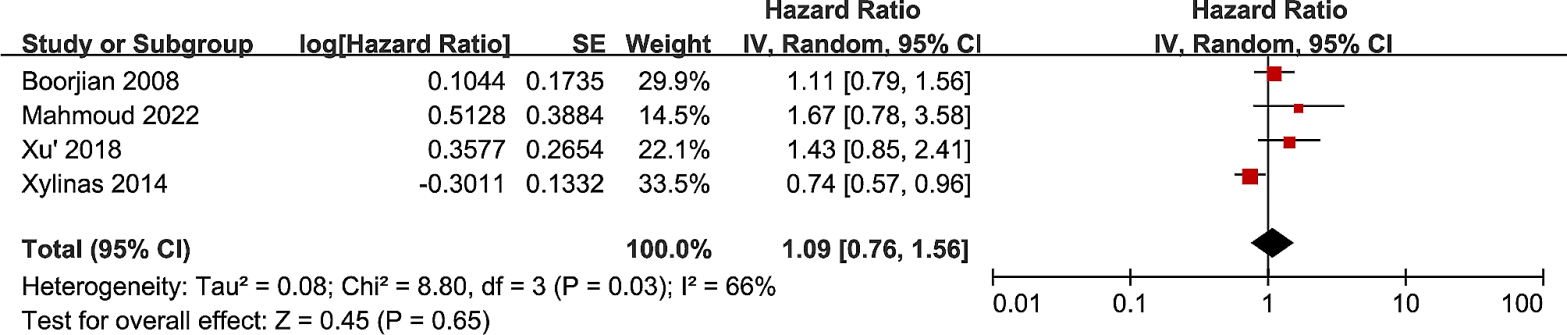
Forest plot of the association between B7-H3 expression and OS

Five studies explored the association between B7-H3 expression and patient survival, with four studies calculating BC’s OS [33, 35, 36, 38], and two studies reporting MIBC’s OS [34, 35]. The combined HR of the first four articles was 1.09 (95% CI 0.76–1.56; *P* = 0.65; I2 = 66%, *P* = 0.03; Fig. 5), suggesting no significant correlation between B7-H3 expression and overall survival (OS) in bladder cancer patients. However, due to the limited number of studies and available data, further evaluation of the association between B7-H3 expression and PFS was not feasible.

### Sensitivity analysis

Slight heterogeneity was found between B7-H3 expression and overall survival (*P* = 0.03). When the study of Xylinas 2014 et al [33] was removed, the recalculated hazard ratio (HR) was 1.25 (95% CI = 0.96–1.63, *P* = 0.1), and no heterogeneity was present (*P* = 0.53). Significant heterogeneity was found between bladder cancer and nontumor controls (*P* < 0.00001). By sequentially removing two studies - Boorjian 2020 et al [38] and Xylinas 2014 et al [33] - the pooled odds ratio (OR) was recalculated to be 9.36 (95% CI = 5.78–15.16, *P* < 0.00001), with no heterogeneity (*P* = 0.82).

### Publication bias

**Fig. 6:**
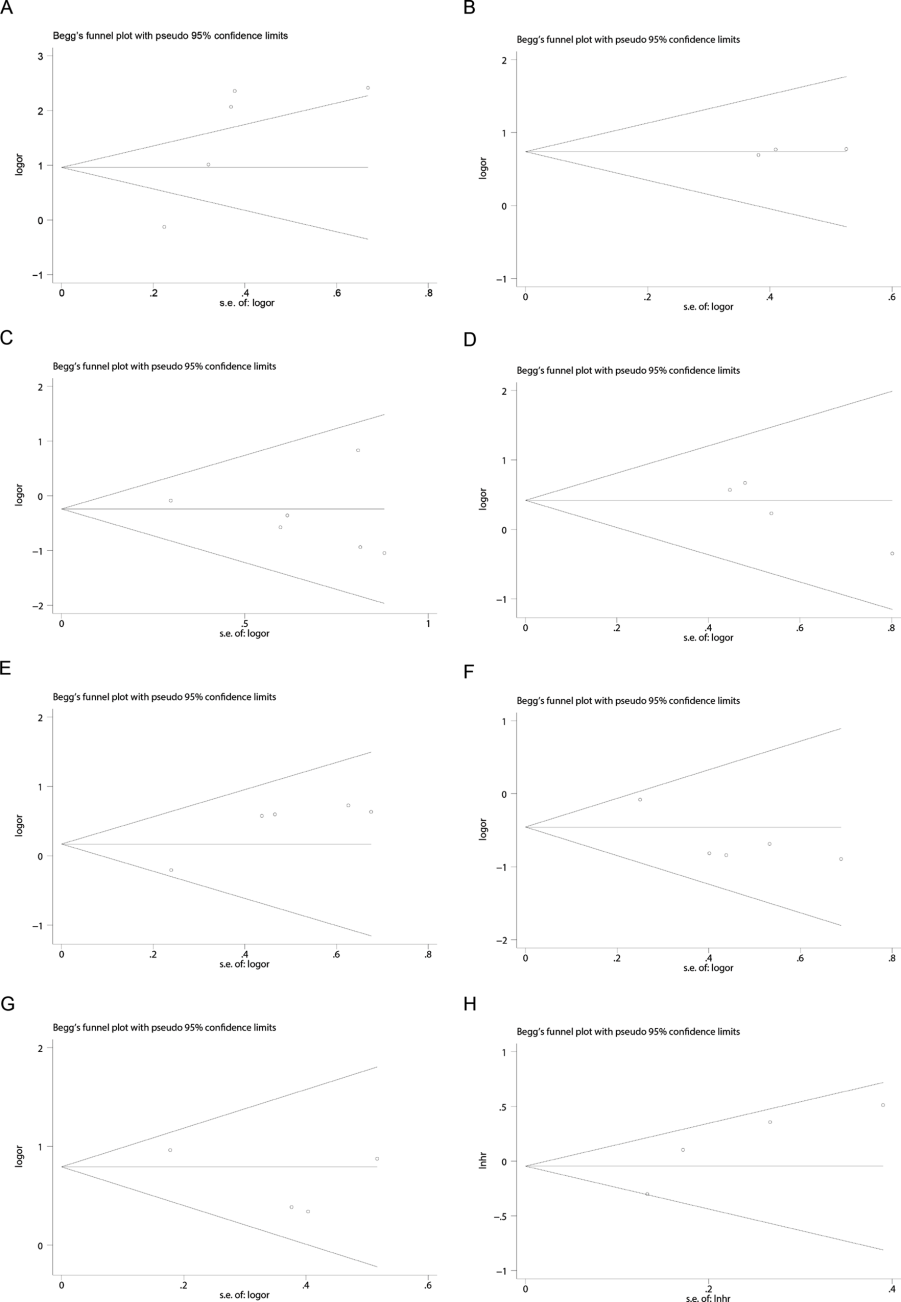
Funnel plot of studies assessing publication bias of the relationship between B7-H3 expression and **A** age, **B** gender, **C** tumor grade, **D** lymph node metastasis, **E** depth of invasion, **F** recurrence, **G** prognostic significance

**Fig. 7:**
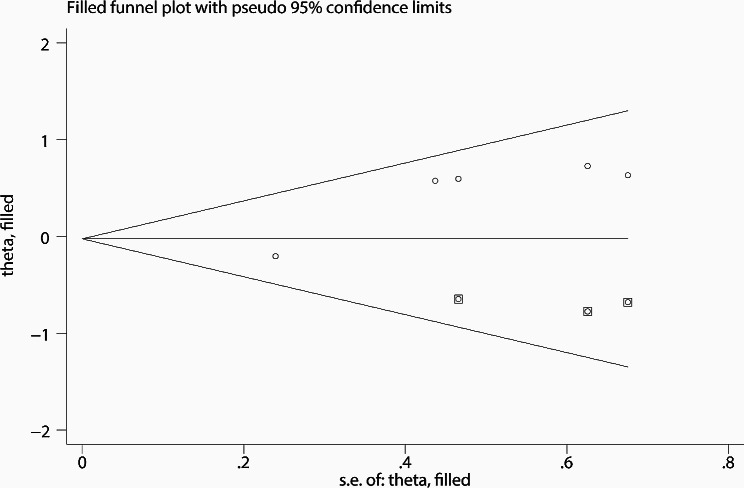
Funnel plot of studies assessing publication bias of the relationship between B7-H3 expression and lymph node metastasis after trim and fill method

The funnel plot obtained from the Begg test is shown in Fig. 6, and Table 3 lists the corresponding Egger test and Begg test p-values. The correlation analysis of cancer B7-H3 expression with age, gender, T stage, tumor grade, recurrence, or overall survival did not demonstrate significant publication bias (*P* > 0.05). However, there was some evidence of publication bias in the analysis of cancer B7-H3 expression and lymph node metastasis (*P* < 0.05). Therefore, trimming and populating analyses were performed to overcome publication bias. After filling three studies, the populated dataset showed no evidence of publication bias (Fig. 7). The new dataset moved the estimated combined OR from 1.20 (95% CI:0.85–1.68) to 0.98 (95% CI:0.72–1.32).

**Table 3:** Evaluations for the potential publication bias within the meta-analysis

Groups of outcomes	Studies(n)	Estimates	Begg’s test (p value)	Egger’s test (p value)	Publication bias
Age(≥ 65 vs. <65 years)	3	OR with 95% CI	1.000	0.517	Not significant
Gender(Male vs. Female)	6	OR with 95% CI	0.260	0.498	Not significant
Tumor grade(low vs. high)	4	OR with 95% CI	0.308	0.060	Not significant
LN metastasis(Positive vs. negative)	5	OR with 95% CI	0.806	0.021	Significant
Tumor stage(Ta-2 vs. T3-4)	5	OR with 95% CI	1.000	0.066	Not significant
Recurrence vs. No recurrence	4	OR with 95% CI	0.734	0.277	Not significant
Prognostic significance	5	HR with 95% CI	0.308	0.112	Not significant

OR odds ratio; HR hazard ratio; CI confidence interval

## Discussion

Bladder cancer (BC) represents a global health challenge with high morbidity and mortality rates [1]. Despite the advancements in biotechnology, numerous prognostic markers have been identified for bladder cancer; however, each comes with its own set of limitations [13, 15, 21]. Consequently, there is an imminent demand for the identification of a superior marker.

B7-H3, also known as CD276, belongs to the B7 family of immune checkpoint proteins. It is an immunoglobulin type I transmembrane protein composed of 316 amino acids. In humans, it is encoded by chromosome 15q24 and consists of an extracellular structural domain, a transmembrane structural domain, and a short intracellular tail [40]. While B7-H3 mRNA is expressed in most normal tissues, B7-H3 protein is either not expressed or expressed at very low levels in normal tissues [41, 42]. However, it is highly expressed in various cancer cells, including breast [43], lung [44], and ovarian cancers [45]. With the advancement of research, accumulating evidence has highlighted a strong association between B7-H3 and the initiation and progression of different cancers. Aberrant B7-H3 expression has been implicated in influencing multiple cellular processes, such as migration, invasion, chemoresistance, endothelial-to-mesenchymal transition, and tumor cell metabolism [26, 46–51]. Furthermore, several studies [24, 27, 52, 53] have reported that patients with elevated tumor B7-H3 levels experienced shorter survival times.

The role of B7-H3 in the prognosis and clinicopathological features of bladder cancer (BC) has been investigated in several studies, but the results have shown inconsistencies [33, 35, 36, 38]. Therefore, we conducted this meta-analysis, including eight articles encompassing a total of 1622 patients. Our findings indicated that B7-H3 expression was not associated with a poor prognosis in BC patients, while B7-H3 expression levels were higher in BC patients compared to normal tissues. In addition to investigating the prognostic aspect, we also analyzed the relationship between B7-H3 and clinicopathological features of BC. The results revealed a significant association between high B7-H3 expression and age, tumor infiltration, and recurrence. However, no significant relationship was observed between B7-H3 and other clinical characteristics. It is worth noting that our data did not support the idea that B7-H3 expression predicts poor survival in bladder cancer. This discrepancy may be attributed, in part, to the limited total sample size in our meta-analysis, which could introduce bias.

Currently, the mechanisms underlying B7-H3’s role in tumorigenesis and development remain unclear. However, several potential mechanisms have been proposed. Firstly, B7-H3 affects tumor cell metabolism, as evidenced in triple-negative breast cancer where decreased B7-H3 expression reduces tumor cell glycolytic capacity and increases sensitivity to AKT/mTOR inhibitors [46]. Secondly, B7-H3 suppresses T-cell-mediated antitumor immunity by inducing M2-type polarization of tumor-associated macrophages in hepatocellular carcinoma, thereby promoting tumor development through the STAT3 signaling pathway [54]. Thirdly, B7-H3 plays a role in tumor promotion by influencing cell proliferation, metastasis, invasion, and epithelial-to-mesenchymal transition (EMT). For instance, B7-H3 knockdown in esophageal carcinoma and breast cancer suppresses tumor cell proliferation [47, 48], while in pancreatic cancer and gastric cancer, it reduces tumor cell migration and transwell invasion in vitro [49]. Furthermore, B7-H3 promotes EMT in glioma and hepatoma cells through JAK2/STAT3/Slug pathway activation [26, 50]. Lastly, B7-H3 enhances chemoresistance, as shown in human pancreatic ductal adenocarcinoma cells where B7-H3 silencing increases sensitivity to gemcitabine. These mechanisms provide valuable insights into the diverse roles of B7-H3 in cancer development and progression, warranting further investigation to fully elucidate its functions and potential therapeutic implications [51].

However, the assessing standard of B7-H3 positivity is different in different studies. Boorjian [38], Xylinas [33] and Li [37] considered cases with B7-H3 cell staining ≥ 10% as positive, in addition Li [37]subdivided B7-H3 positivity, with 10–29% defined as low expression, 30–60% defined as moderate expression and > 60% defined as intense expression. However, these methods are based on staining area only, on which Mahmoud [36] and Xu [32, 34, 35] also considered staining intensity. In Xu’ s study [32, 34, 35], quantification was made as follows: <33% of cancer cells—1; ≥33 to 66% of cancer cells—2; >66% of cancer cells—3; absent/weak staining—1; moderately intense staining—2; strong staining—3. By multiplying the above two scores, a total score of ≤ 3 was defined as low expression of B7-H3, and a total score of > 3 was defined as high expression of B7-H3. And Mahmoud [36] use H-score, which is equal to 1×(% cells weak expression) + 2×(% cells with moderate expression) + 3×(% cells strong expression). The groupings are as follows: negative (H-score = 0); Low (H-score ≥ 1 and < 120); high (H-score ≥ 120). Further research is needed on which assessment criteria are better.

In recent years, immunotherapy has made rapid advancements in the treatment of various types of cancer. Progress in molecular biology and antibody engineering has facilitated the development of strategies for targeting B7-H3 using multiple effector mechanisms. Many of these approaches have been successfully tested in vitro and in mouse models, demonstrating encouraging safety profiles and anti-tumor activity [55–58]. These results have paved the way for clinical trials focusing on B7-H3 targeting. In the context of bladder cancer, Ma et al. [59] demonstrated an increase in CD69 expression on activated T cells (ATC) when treated with an anti-CD3-B7-H3 bispecific antibody (B7-H3Bi-Ab). CD69 is considered an early activation marker for T cells. B7-H3Bi-Ab-ATC have the capability to eliminate B7-H3-positive bladder cancer cells through the CD3-B7-H3 bridge mechanism, exhibiting significant cytotoxic activity against human bladder cancer cells. These findings suggest that B7-H3Bi-Ab enhances the killing potential of ATC against bladder cancer cells, offering a promising novel approach for the treatment of bladder cancer.

Although efforts have been made, our study still has some limitations. Firstly, the main ethnic populations were European and Asian, and African representation was insufficient. Secondly, the pooled analyses of the relationships between B7-H3 expression and prognosis and patient clinicopathological features, such as tumor grade and T stage, were based on a relatively small number of studies. More research is needed to validate these results and obtain more reliable conclusions. Thirdly, the included studies used different criteria for judging the positive or negative expression of B7-H3, leading to some heterogeneity in the meta-analysis. This could affect the overall accuracy of the results. Fourthly, we did not include unpublished articles and conference abstracts in the meta-analysis due to insufficient information, which may introduce selection bias and potentially miss relevant data. Fifthly, the sample size of our meta-analysis was relatively small, which may limit the statistical power to detect certain associations, especially in the analysis of overall survival. Lastly, because some included articles did not report detailed survival data, we had to rely on the Kaplan-Meier curve for survival analysis, which might lead to potential overestimation or underestimation of actual survival data. To address these limitations, future research should focus on conducting well-designed clinical randomized controlled studies with larger sample sizes that encompass diverse racial backgrounds to provide more robust and comprehensive insights into the relationship between B7-H3 expression and bladder cancer.

## Conclusion

In conclusion, our study confirms that B7-H3 is overexpressed in bladder cancer (BC), and its expression is closely related to tumor invasion and recurrence in BC. Furthermore, B7-H3 expression has shown no association with the survival of bladder cancer patients, suggesting that B7-H3 may play multiple roles in the pathophysiology of bladder cancer. To address the limitations of the current meta-analysis, it is imperative that future studies with larger sample sizes and standardized methodologies be conducted. Additionally, investigating the underlying mechanisms by which B7-H3 influences tumor progression holds the potential to contribute to the development of innovative treatment strategies for bladder cancer.

## Data Availability

Data come from published literature.
